# Predictors of adherence to antiretroviral therapy among HIV-infected persons: a prospective study in Southwest Ethiopia

**DOI:** 10.1186/1471-2458-8-265

**Published:** 2008-07-30

**Authors:** Alemayehu Amberbir, Kifle Woldemichael, Sofonias Getachew, Belaineh Girma, Kebede Deribe

**Affiliations:** 1 Butajira Birth Cohort Study, School of public health, Addis Ababa University, P.O.Box 80596, Addis Ababa, Ethiopia; 2 Department of Epidemiology and Biostatistics, Faculty of public health, Jimma University, Jimma, Ethiopia; 3 ICAP International Ethiopia, Addis Ababa, Ethiopia; 4 Addis Continental Institute of public health, Addis Ababa, Ethiopia; 5 Fayyaa Integrated Development Association- NCMI, PEPFAR-New Partners, Initiative: Program Director, Addis Ababa, Ethiopia

## Abstract

**Background:**

The devastating impact of AIDS in the world especially in sub-Saharan Africa has led to an unprecedented global effort to ensure access to antiretroviral (ARV) drugs. Given that medication-taking behavior can immensely affect an individual's response; ART adherence is now widely recognized as an 'Achilles heel' for the successful outcome. The present study was undertaken to investigate the rate and predictors of adherence to antiretroviral therapy among HIV-infected persons in southwest Ethiopia.

**Methods:**

The study was conducted in the antiretroviral therapy unit of Jimma University Specialized Hospital. A prospective study was undertaken on a total of 400 HIV infected person. Data were collected using a pre-tested interviewer-administered structured questionnaire at first month (M_0_) and third month (M_3_) follow up visits.

**Results:**

A total of 400 and 383 patients at baseline (M_0_) and at follow up visit (M_3_) respectively were interviewed. Self-reported dose adherence in the study area was 94.3%. The rate considering the combined indicator (dose, time and food) was 75.7%. Within a three month follow up period, dose adherence decreased by 2% and overall adherence rate decreased by more than 3%. Adherence was common in those patients who have a social support (OR, 1.82, 95%CI, 1.04, 3.21). Patients who were not depressed were two times more likely to be adherent than those who were depressed (OR, 2.13, 95%CI, 1.18, 3.81). However, at the follow up visit, social support (OR, 2.42, 95%CI, 1.29, 4.55) and the use of memory aids (OR, 3.29, 95%CI, 1.44, 7.51) were found to be independent predictors of adherence. The principal reasons reported for skipping doses in this study were simply forgetting, feeling sick or ill, being busy and running out of medication in more than 75% of the cases.

**Conclusion:**

The self reported adherence rate was high in the study area. The study showed that adherence is a dynamic process which changes overtime and cannot reliably be predicted by a few patient characteristics that are assumed to vary with time. Adherence is a process, not a single event, and adherence support should be integrated into regular clinical follow up.

## Background

An estimated 33.2 million people worldwide were living with HIV, 2.5 million became newly infected and 2.1 million lost their lives to AIDS at the end of 2007 [1]. Sub-Saharan Africa remains the worst affected region in the world. A little more than one-tenth of the world's population lives in sub-Saharan Africa, which is home to almost 68% of all people living with HIV [1]. The HIV/AIDS epidemic in Ethiopia continues to pose a threat to the lives of its people. It is estimated that 977,394 people live with the virus resulting in 71,902 HIV related deaths in 2007 [2]. The national prevalence of HIV in 2007 is estimated to be 2.1% [2].

Highly Active Antiretroviral Therapy (HAART) was a breakthrough in the industrialized world, leading to the reduction of mortality and the improvement of quality of life of people living with HIV and AIDS (PLWHA) [3,4]. It transformed the disease into a chronic treatable condition for a significant proportion PLWHA with access to this treatment [5]. The Government of Ethiopia introduced the ART program with the goal to prolong the lives, to restore the mental and physical functions and to improve the quality of life of PLWHA [6]. ART was first offered in July 2003 through 12 government hospitals on a co-payment basis. In early 2005, 211,000 men, women and children needed ART but only 16,400 were receiving it. In January 2005, free ART through the Global Fund, World Bank and PEPFAR (US President's Emergency Plan for AIDS Relief) became available in 22 hospitals [7].

Even though ART is the single most dramatic development yet in the treatment of HIV [8], many have been described as being inconsistent with their treatment regimens, either not taking prescribed medication, taking medications only when they felt up to it, or needing breaks [9]. ART adherence is now widely recognized as a critical health promotion behavior for HIV positive individuals on therapy [8] and it is the 'Achilles heel' of successful outcome [10]. Adherence to HIV treatment regimen is defined as taking pills in all the prescribed doses at the right time, in the right doses and in the right way [11].

Adherence is the second strongest predictor of progression to AIDS and death, after CD4 count [12]. Consistently high levels of adherence are also important determinants of virologic and immunologic outcome, AIDS-related morbidity, mortality, and hospitalizations [12-16]. Non-adherence risks the development of drug resistance and failure of therapy [17,18]. Although the minimum threshold of adherence necessary for the clinical effectiveness of HAART remains unclear, available data suggests that patients must take a high proportion (95% or more) of antiretroviral drug doses to maintain suppression of viral replication, that failure rates increase as adherence levels decrease [19].

### Predictors of adherence

Studies report conflicting evidence about the association between socio-demographic factors and adherence behavior. Some literatures reported that certain socio-demographic variables have influence over adherence to HAART; however, others showed no association [4,8,13,14,20,21]. More consistent associations are found between certain psychosocial factors and adherence behavior. Common predictors of non-adherence include depression/psychiatric morbidity [23,24], active drug or alcohol use [25], sero status disclosure [24,25] and lack of social support [26].

The complexity of the regimen, side effects and various demands around medication and food timing caused by it are also associated with non-adherence [15,16,27]. However, Gao. X and *et al *showed that regimen complexity alone was not a significant predictor of patients' medication adherence [22]. Various studies have documented that inadequate knowledge and negative beliefs about HIV disease and treatment effectiveness present an important barrier to ART adherence [14,17,24]. Few studies describe a relationship between HIV-related symptoms and non-adherence. Patients who have experienced AIDS-related symptoms perceived as serious are usually more adherent than patients who never had symptoms, or who consider their symptoms unimportant [22,28,29].

As the world gears toward increasing access to antiviral treatment in the developing world it is critical to understand factors (motivators and barriers) that influence adherence to Antiretrovirals and apply the lessons learnt in improving existing and new programs. Available research in Ethiopia has shown that our understanding of factors associated with ART adherence is limited, and related literature in the study area is remarkably scarce. Understanding the predictors of adherence in the local context is a forefront agenda in Ethiopia, where little is known and scaling up of ART program is in progress. In view of this, and to assess whether the global experience works with the Ethiopian context, a prospective study was conducted in southwest Ethiopia to determine the rate, barriers, and factors associated with ART adherence. It is anticipated that the findings generated from this study will contribute to the knowledge and understanding of non-adherence to ARVs and be useful in developing evidence based interventions that are undertaken to address ARV adherence in Ethiopia.

## Methods

### Study setting

The study was conducted in the antiretroviral therapy unit of Jimma University Specialized Hospital (JUSH) from December 25/2006 – April 30/2007 for a period of 4 months. The zone is one of 17 zones in Oromia Regional State; the capital Jimma, is located 335 KM Southwest of Addis Ababa. The Hospital and ART units launched their service as of July 2003 and till recently a total of 2538 PLWHAs were receiving care, of whom 1118 patients were on ART until the end of July 2007 [30]. The study has two phases. Phase I measured adherence at baseline (M_0_), and was conducted from December 25/2006. In phase II the subjects were followed prospectively for three months and self reported adherence was measured at 3^rd ^month (M_3_) starting from March 25/2007.

### Participants

A prospective study was undertaken to investigate the possible factors for adherence. The sample size was calculated using Epi-Info 3.3.2 statistical software, assuming the following parameters. The proportion of adherence among non-depressed individuals was 83.6% with a relative risk of 2.8 [4]. Other parameters include 95% CI and 80% power. Ten percent (10%) of the sample was added to recompense for the loss to follow up and losses due to death. The actual size of the cohort needed for the study was 403. In phase I of the data collection individuals within the intended study period were included until the required sample size was attained. In the II phase, the same subjects were included at third months of their follow up visit.

### Measurement

The dependent variable was adherence to antiretroviral therapy. Adherence in this study was measured by (1) Self-reported dose adherence, defined as patient's self-report of whether any antiretroviral medication had been skipped that day, the previous day, the previous three days or the previous seven days. A person was said to be adherent if he/she took ≥ 95% of the prescribed doses correctly. For the comparison assessment, we used adherence in the previous seven days. (2) Self-reported time adherence; where a person is said to be adherent when claiming to always follow scheduling instructions. (3) Self-reported food adherence; where a person is said to be adherent when always following dietary instructions agreed upon with the providers. Hence, for comparison purposes a combined indicator of adherence was made using the three adherence measures taking into account all questions pertaining adherence. Accordingly a person was said to be adherent when he/she took >= 95% of the prescribed doses correctly, always followed scheduling instructions and always followed dietary requirements. This type of measurement of adherence has been frequently used in a range of studies [31-33].

Data were collected using a pre-tested questionnaire which consists of socio-demographic characteristics (e.g. age, sex, education, occupation, income, marital status, ethnicity, address), psychosocial attributes (e.g. social support, depression, active substance and alcohol use, disclosure of HIV sero status, use of memory aids, HIV/AIDS stigma), disease characteristics (WHO clinical staging, duration of HIV infection), regimen related variables (dosing schedules and frequency, pill burden and complexity, dietary related demands, side effect, history of hospitalization), health care system and health care team related variables (ongoing care and follow up, convenience of schedules and appointments).

Depression was measured using a 13 item scale widely used in the HIV/AIDS literature, after excluding items reflecting somatic symptoms. Accordingly, a cut-off point of 10 was used in this study to differentiate depressed from non-depressed individuals [34]. Stigma was measured using items drawn from a previous scale [35]. The total HIV/AIDS stigma scale was measured using a 23 item questionnaire with a score ranging from 23 to 92. A person is said to be stigmatized when he/she scored above the mean.

### Data analysis

The data collected from the respondents were cleaned, coded, entered and analyzed using SPSS 12.0.1 for Windows at baseline (M_0_) and repeat (M_3_) visits. Self-reported dose adherence to all antiretroviral agents was summarized as the ratio of the average daily number of antiretroviral medications adhered to correctly according to the standard instructions over the total number of antiretroviral medication prescribed. The analysis consisted of basic summaries of patient characteristics, and bivariate analysis of the relation between adherence and various factors. The reliability of scale items was evaluated using Cronbach's alpha; which measures how well each individual item in a scale correlates with sum of the remaining items. A cut-off value of 0.7 was used to indicate acceptable internal consistency [36]. Two logistic regression models were performed with adherence (to dose, schedule and food) at M_0 _and M_3 _as the dependent variable to determine the constant predictors. In both cases, all explanatory variables that were associated with the outcome variable (adherence at M_0 _and M_3_) in the bivariate analyses with a P-value of 0.05 or less were included in the initial logistic models. The models were evaluated using a forward and backward stepwise elimination procedure. A P-value of 0.2 was used to select variables for entry into the model and 0.1 for removal from the model.

### Human subjects

The study was approved by the Institutional Ethical Review Committee (IERC) of Jimma University.

## Result

### Sociodemographic characteristics

Patients who came to the ART unit during the first phase of the data collection were included in the baseline study (M_0_). A total of 403 subjects gave informed consent and were interviewed, of which three had missing adherence data and were omitted from the analysis. At the follow up visit (M_3_) a total of 383 subjects completed the study with a median length of follow up and inter-quartile range of 90 (88–92) days. The remaining 17 subjects were not present at the third data collection phase and were not included in the follow up study.

The subjects' age ranged from 19 to 58 years with a median age of 30 years. Most were females which accounted for 239 (59.8%). The majority 389 (97.3%) were from Jimma, were Oromo 161 (40.3%) by Ethnicity and Orthodox 231 (57.8%) by religion. One hundred eighty (45%) were married, 201 (50.3%) had attended secondary education, 143 (35.8%) had no job and 148 (37%) of the survey participants had no monthly income. Three hundred twelve (80.7%) of the study subjects were living with their husband/wife, family or their friends and only 77 (19.3%) lived alone (Table 1).

**Table 1:** Socio-demographic characteristics of the study participants at baseline (M_0_) in JUSH, Southwest Ethiopia, 2007 (N = 400)

Characteristics	Fr^b ^(%)
Sex	
Male	161(40.3)
Female	239(59.8)
Age	
18–24	54(13.5)
25–34	194(48.5)
35–44	120(30.0)
≥ 45	32(8.0)
Permanent address	
Jimma	389(97.3)
Out of Jimma	11(2.8)
Ethnicity	
Oromo	161(40.3)
Amhara	126(31.5)
Kefa	35(8.8)
Dawro	31(7.8)
Gurage	21(5.3)
Tigre	12(3.0)
Others*	14(3.3)
Marital Status	
Married	180(45.0)
Single	78(19.5)
Windowed	72(18.0)
Separated	41(10.3)
Divorced	29(7.3)
Religion	
Orthodox	231(57.8)
Muslim	99 (24.8)
Protestant	62 (15.5)
Others**	8(2.0)
Educational status	
Illiterate	43(10.7)
Read & write/no formal education	14(3.5)
Elementary	110(27.5)
Secondary	201(50.3)
12^+^	32(8.0)
Occupation	
Employed	139(34.8)
Merchant	68(17.0)
Daily laborer	36(9.0)
Have no job	143(35.8)
Others^£^	14(3.5)
Monthly income ETB^a^	
≤ 500	196(78.1)
501–999	38(15.1)
≥ 1000	17(6.8)

*Yem, Wolayita, Kenbata, Sidama, Bench, **Catholic, Jova whiteness, No religion ^£^Farmer, Prisoner, Bar lady, Retired

a Exchange rate 1 USD = 8.6 Ethiopian Birr (ETB), b Frequency

### Clinical Markers

The majority of the subjects, 265 (66.3%) had started HAART while at WHO disease stage III. In addition for those patients in which the initial CD_4 _count was done, the majority 270 (72.2%) had CD4 count less than 200 cells/mm^3^; the range being 2 to 749 cells/mm^3 ^with a median of 135 cells/mm^3^. The study subjects, at inclusion, were on HAART for a median duration of 8 months (3 to 67 month). Most 384 (96%) of them had monthly regular follow up visit for their drug refill (Table 2).

**Table 2:** Clinical markers of the study participants at baseline (M_0_), JUSH, Southwest Ethiopia, 2007.

Characteristics	Fr (%)
WHO disease stage when HAART started (N = 400)	
I	3(.8)
II	33(8.3)
III	265(66.3)
IV	99(24.8)
CD_4 _count when the treatment was started (N = 374)	
≥ 500	3(.8)
200–499	101(27.0)
< 200	270(72.2)
Duration of treatment in months (N = 400)	
3–12	279(69.8)
13–24	112(28.0)
≥ 25	9(2.3)
Clinical Follow Up (N = 400)	
Monthly	384(96.0)
Every two month	15(3.8)
Every three month	1(.3)

### Psychosocial and disease characteristics

Among the psychosocial characteristics of the study subjects, the majority 311 (77.8%) received social support at baseline (M_0_) either from their family, friends/peers, HIV clubs or co-workers. The study subjects reporting social support decreased to 207 (54%) at M_3 _the difference being significant (P ≤ 0.05). Using the HIV/AIDS stigma scale (Cronbach's alpha = 0.94) at M_3 _121 (32.2%) of the study subjects were stigmatized. Thirty eight (9.5%) of participants at M_0 _reported active substance use. However, 39 (10.2%) reported active substance use at M_3 _(P > 0.05). Based on the Beck's depression inventory (BDI) scale with a cut-off point of ten, 223 (55.8%) and 190 (49.6%) of the study subjects were depressed at baseline and follow up visit respectively (P > 0.05). Side effects were experienced by 209 (52.3%) of the patients at baseline. However, after 3-month follow up period only 78 (20.4%) reported having side effects (P > 0.05). Twenty eight (7%) of the study subjects at baseline were hospitalized after they started HAART. Of these, 24 (88.9%) were hospitalized at least once; the rate being 2 times higher among non adherent than adherent individuals. Hospitalization was further decreased at M_3 _in which only 8 (2.1%) had a history of admission (p > 0.05). Three hundred ninety five (98.8%) and 343 (89.6%) of the study participants reported using memory aids at M_0 _and M_3 _respectively (Table 3).

**Table 3:** Psychosocial and disease related characteristics of the study participants at baseline (M_0_) and follow up visit (M_3_) in JUSH, Southwest Ethiopia, 2007 (N_1_* = 400, N_2_* = 383).

Characteristics	Baseline (M_0_)	Follow up (M_3_)	P-value
	
	Fr(%)	Fr(%)	
Memory aids			
Yes	395(98.8)	343(89.6)	0.087^†^
No	5(1.3)	40(10.4)	
Social support			
Yes	311(77.8)	207(54.0)	0.027
No	89(22.3)	176(46.0)	
Active substance use ((N_1_£)			
Yes	38(9.5)	39(10.2)	0.23
No	361(90.5)	344(89.8)	
Depression (BDI > 10^φ^)			
Yes	223(55.8)	190(49.6)	0.43
No	177(44.3)	193(50.4)	
Side effect			
Yes	209(52.3)	78(20.4)	0.14
No	191(47.8)	305(79.6)	
Hospitalization after treatment (N_1_£)			
Yes	28(7.0)	8(2.1)	0.45^†^
No	371(92.8)	375(97.9)	

*No of cases at M_0 _and M_3_, **three cases were missed, ‡two cases were missed, £ one case was missed, ^φ^Beck's depression inventory, ^†^Fisher's exact test.

### Adherence rate and Reasons for non adherence

**Figure 1:**
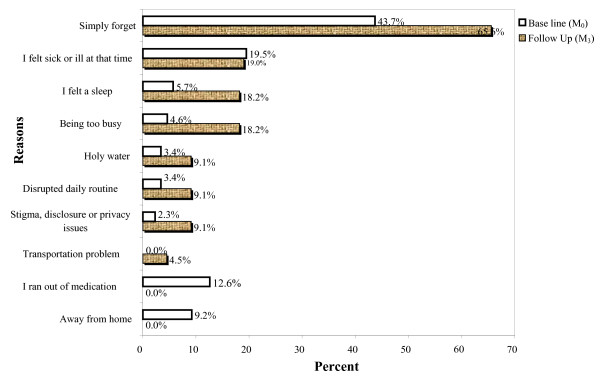
Reasons for skipping doses at base line (M_0_) and follow up visit (M_3_) in JUSH, Southwest Ethiopia 2007.

The three adherence errors were assessed in the study to get a combined adherence indicator (Table 4). Accordingly, 384 (96%) and 361(94.3%) of the study subjects were adherent based on self-report of missed doses (dose adherence) in a one-week recall at M_0 _and M_3 _respectively (P = 0.54). Three hundred eighty nine (97.2%) and 373 (97.4%) of the study subjects always followed the time restrictions (time adherence) agreed upon with their providers at M_0 _and M_3 _respectively (P = 0.77). Three hundred thirty eight (84.5%) and 319 (83.3%) subjects followed instructions related to food (food adherence) all the time at M_0 _and M_3 _respectively (P = 0.79). Hence, the rate of self reported adherence in the study area based on the combined indicator of the three adherence errors was 79.3% at baseline and 75.7% at follow up visit (P = 0.62) (table 4). The principal reasons reported for skipping doses were similar for both visits. At baseline most 38 (43.7%) simply forget, 17 (19.5%) felt sick or ill at that time, and 11 (12.6%) ran out of medication. During the follow up visit again the majority 14 (65.6%) simply forgot, 4 (19%) felt sick and 4 (18%) were busy (Fig. 1).

**Table 4:** Self reported adherence by adherence categories (dose, time and food) for the study subjects at base line (M_0_) and follow up visit (M_3_) in JUSH, Southwest Ethiopia, 2007.

Adherence category	Baseline (M_0_)	Follow up Visit (M_3_)	P-value
		
	Fr (%)	Fr (%)	
Self reported 7-day recall dose adherence (N_1_* = 400, N_2_* = 383)			
Adherent	384(96.0)	361(94.3)	0.54^£^
Non- adherent	16(4.0)	22(5.7)	
Self reported time adherence (N_1 _= 400, N_2 _= 383)			
Adherent	389(97.2)	373(97.4)	0.77^£^
Non- adherent	11(2.8)	10(2.6)	
Self reported food adherence (N_1 _= 400, N_2 _= 383)			
Adherent	338(84.5)	319(83.3)	0.79
Non- adherent	62(15.5)	64(16.7)	
Adherence to all (Dose, Schedule and Food) (N_1 _= 400, N_2 _= 383)			
Adherent	317(79.3)	290(75.7)	0.62
Non- adherent	83(20.8)	93(24.3)	

*N_1 _total number of cases at baseline (M_0_) and N_2 _at follow up (M_3_), ^£^Fisher's Exact Test

### Predictors of adherence

In the multivariate logistic regression analysis; three variables were found to be predictors of adherence at baseline (M_0_). Adherence was common in those patients who have a social support (OR, 1.82, 95%CI, 1.04, 3.21). Patients who were not depressed were two times more likely to be adherent than those who were depressed (OR, 2.13, 95%CI, 1.18, 3.81). However, at the follow up visit, social support (OR, 2.42, 95%CI, 1.29, 4.55) and the use of memory aids (OR, 3.29, 95%CI, 1.44, 7.51) were found to be independent predictors of adherence (Table 5).

**Table 5:** Multivariate logistic regression analysis: Variables that predict adherence to dose, time and food at baseline (M_0_) and follow up visit (M_3_) in JUSH, Southwest Ethiopia, 2007.

	**Model 1**			**Model 2**		
	**At baseline (M_0_)**			**At follow up visit (M_3_)**		
	
Variables	Adherence Yes n(%)	**Adjusted OR^† ^(95% CI)**	P-value	Adherence Yes n(%)	**Adjusted OR^† ^(95% CI)**	P-value
Social support						
Yes	259(83.3)	1.82(1.04,3.21)	0.038	168(81.2)	2.42(1.29,4.55)	0.006
No	58(65.2)	1		122(69.3)	1	
Depression						
Yes	160(71.7)	1	0.011	***NA***		
No	157(88.6)	2.13(1.18, 3.81)				
Memory aids						
Yes	***NA***			268(78.1)	3.29(1.44, 7.51)	0.005
No				22(55.0)	1	

^†^Adjusted for all socio-demographic, psychosocial, regimen and disease related variables. ^†^adjusted for baseline socio-demographic characteristics. NA = not applicable in the respective regression models.

## Discussion

In most studies, adherence refers solely to dose adherence, but successful treatment with ART also includes adhering to scheduling and adhering to dietary instructions that accompany many antiretroviral drugs [32,33]. In this study we assessed scheduling and dietary instructions as two additional independent types of adherence and a combined indicator was made to determine the rate of adherence in the study area. Participants' self-reports of adherence in this study indicated a high degree of dose and scheduling adherence, while occasional suboptimal adherence of dietary instructions was quite common.

The rate of dose adherence in the study area was 96% at baseline and 94.3% at M_3_; which is higher that reported in Addis Ababa and Arbaminch, Ethiopia [4,37]. Consistent findings were also documented in comparable studies in resource limited settings in the sub-Saharan Africa [38]. The overall rate of self reported adherence in the study area based on the combined indicators of the three adherence errors was 79.3% at base line and 75.7% at M_3_. Some studies in resource-rich settings have documented less than 50% of patients taking all their antiretroviral medications on time and according to dietary instructions [31,33]. This was much lower than our report confirming that patients in developing countries can achieve good adherence despite limited resources. Orrell *et al *also found that low socio economic status was not a predictor of adherence for patients with fully subsidized therapy and concluded that adherence in developing countries has been found to be at least as good as adherence in developed countries [39].

This relatively short prospective study underscores the dynamic nature of adherence. Though not significant, within three months, dose adherence decreased by 2%, food adherence by 1% and overall adherence rate reduced by more than 3%. The lack of statistical significance difference may be due to the short duration of follow up. Various studies have indicated the dynamic nature of adherence overtime [31,33,40]. Thus, we believe that the continual monitoring of adherence rate and its determinants in Ethiopian should not be undermined and require further study.

The principal reasons reported for skipping doses were similar to other studies at both visits [4,37,38]. The most important reasons our participants cited were simply forgetting, feeling sick or ill, being busy and running out of medication in more than 75% of the cases. This study shows that patients have a range of reasons for failing to adhere to their antiretroviral regimens. These reasons should be assessed for an individual patient and appropriate adherence-enhancing intervention should be undertaken. In this case, adherence counseling might incorporate strategies to avoid simply forgetting taking pills like the use of memory aids.

Researches' have already shown the dynamic character of HAART-treated patients' adherence behaviors, which are influenced by multiple factors varying over time [31,33]. Our study also showed that even short-term non-adherence cannot be reliably predicted on the sole basis of a few patient characteristics that could vary over time. In line with this, depression was one of the predictor variables which were amenable for intervention. Patients who did not have depressive symptoms were two times more likely to be adherent than depressed one. Similar results were also reported in Addis Ababa, Ethiopia [4]. These findings support a role for HIV/AIDS providers/counselors in screening for depression and providing treatment when appropriate, either directly or through collaboration with mental health professionals. Most importantly the formulation of simple and locally validated screening tool for depression in the Ethiopian context is underscored.

Our results also show that patients who claimed to use memory aids were three times more likely to be adherent than those who did not. This study shows that adherence interventions should include memory aids and other reminders to help patients take their drugs. Social support was a constant predictor of adherence identified in this study. Patients who reported social support were more likely to be adherent than those who did not. This is consistent with many comparable studies both in resource poor and resource rich settings [4,14,18,24,38]. Hence, the initial adherence assessment and preparation should include a discussion on the sources of social support for the individual patient and an attempt should be made for possible solutions prior to starting HAART. Further, enlisting support to help patients take their medications correctly, from the family, community health workers, and PLWHA support groups should also be emphasized.

The findings of this study must be interpreted in the light of its limitations. Because it was conducted at a single site, the findings may not be generalizable to other clinical settings. There is no gold standard for measuring adherence and our measurement of adherence is only based on patients' reports of missed doses, scheduling instructions and dietary requirements. This may be subject to social desirability and recall biases and the literature suggests that patients tend to overestimate adherence [23]. However, many other studies document that well collected self report data clearly correlates with virologic changes and is more practical in most settings [3,43]. We were also unable to relate the obtained adherence rate to viral loads and CD4 cell responses due to financial and logistical barriers to frequent laboratory monitoring in this setting. Further, those subjects who have missed their clinical appointment at the follow up visit may have effect on the outcome of interest. Despite the aforementioned limitations, the prospective design allowed us to assess patient characteristics which are assumed to vary overtime and enabled us to detect the dynamic nature of adherence. Moreover, measurement of adherence was not only based on patients' intake of prescribed doses, but other important dimensions of adherence behavior (with respect to food and timing requirements of prescribed regimens) were examined.

## Conclusion

The adherence rate found in this study seems to be encouraging. The findings emphasized the importance of multiple periodic assessments of adherence errors. Timely detection of non-adherence behaviors and appropriate monitoring of patients' difficulties with HAART could potentially help patients to maintain adherence and therefore improve the treatment outcome. Finally the results suggested that psychosocial and medical interventions aimed at increasing adherence of HAART-treated patients should integrate the dynamic dimensions of adherence behaviors. Adherence is a process, not a single event, and adherence support must, therefore, be integrated into regular clinical follow up. Investigation of factors related with long-term adherence would require longer follow-up than the present study.

## Competing interests

The authors declare that they have no competing interests.

## Authors' contributions

AA conceived and participated in the design, conduct, analysis and interpretation of the study. KW, SG and BG and KD involved in designing the survey and undertook preliminary analysis. All authors contributed to the final report and approved the final manuscript.

## Pre-publication history

The pre-publication history for this paper can be accessed here:


